# Acute kidney injury induced by mixed poisoning with brodifacoum and bromadiolone: a case report

**DOI:** 10.3389/fmed.2025.1667137

**Published:** 2025-12-09

**Authors:** Sha Yang, Yiqian Guo, Qin Yang, Yuting Wang, Hong Chen, Longyin Zhu

**Affiliations:** Department of Nephrology, The First Hospital Affiliated to Army Military Medical University (Southwest Hospital), Chongqing, China

**Keywords:** bromadiolone, brodifacoum, anticoagulant rodenticides, acute kidney injury, obstructive nephropathy, vitamin K1, case report

## Abstract

**Background:**

Anticoagulant rodenticides (ARs) are a significant cause of global poisoning events. AR poisoning primarily manifests as multiorgan hemorrhage, while acute kidney injury (AKI) is exceedingly rare. Current understanding of the pathogenesis of AR-induced AKI remains insufficient, potentially leading to diagnostic and therapeutic delays and increasing the risk of progression to chronic kidney disease. This case report aims to describe the clinical characteristics, diagnostic process, and multidisciplinary treatment strategy of a patient with AKI induced by mixed brodifacoum and bromadiolone poisoning, and to explore the underlying pathogenic mechanisms of AR-induced AKI.

**Case presentation:**

This case report presents a 37-year-old male who developed anuria following the onset of painless macroscopic hematuria and gingival bleeding. Laboratory investigations revealed: prothrombin time (PT) > 120 s, international normalized ratio (INR) 13.58, significantly reduced activity of coagulation factors II, VII, IX, and X, and serum creatinine (Scr) progressively increased to 5.93 mg/dL. Abdominal computed tomography showed bilateral ureteral dilation with hydronephrosis and patchy hyperdense areas in the bladder lumen. Blood toxicology analysis confirmed mixed poisoning with brodifacoum (24.7 ng/mL) and bromadiolone (12 ng/mL). Treatment included intravenous vitamin K1, fresh frozen plasma infusion, cystoscopic evacuation of blood clots and bilateral ureteroscopic double-J stent placement, and continuous renal replacement therapy. At discharge, Scr decreased to 1.52 mg/dL. Ten days post-discharge, follow-up tests showed PT 11.1 s, INR 0.88, and Scr 1.01 mg/dL. Renal function and coagulation profiles remained within normal ranges during follow-up until June 20, 2025.

**Conclusion:**

This case confirms that AR poisoning can induce AKI through a dual mechanism encompassing anticoagulation-related nephropathy and obstructive nephropathy. Implementation of a multidisciplinary strategy successfully achieved renal function reversal, underscoring the importance of early identification of obstructive factors. These findings provide evidence-based guidance for clinical management of such complex cases.

## Introduction

Anticoagulant rodenticides (ARs) are the most widely used agents for rodent control globally, and incidents of poisoning associated with their high toxicity have emerged as a significant public health concern (1–3). While the coagulopathy induced by these toxins has been thoroughly investigated, the pathophysiological mechanisms underlying AR-mediated acute kidney injury (AKI) remain poorly understood. According to the 2019 Annual Report from the American Association of Poison Control Centers’ National Poison Data System (NPDS) (4), approximately 0.5% of human poisoning cases were attributed to rodenticides. Notably, second-generation ARs, including brodifacoum and bromadiolone, significantly increase the risk of fatal poisoning events due to their high lipophilicity and long half-lives (5). In clinical scenarios, poisoning from these substances predominantly presents as multiorgan hemorrhage (6, 7), with reported cases leading to AKI being exceedingly rare. Through a systematic literature search, it was revealed that currently only Wang et al. (8) have reported a case of bromadiolone poisoning that caused coagulopathy, which subsequently led to glomerular hemorrhage and triggered AKI. However, no cases involving urinary obstruction have yet been reported.

Presently, clinical understanding of AKI associated with AR poisoning is limited by two primary limitations: first, most studies attribute the mechanisms of renal injury exclusively to coagulopathy-induced glomerular hemorrhage, overlooking the potentially reversible factor of urinary obstruction due to blood clots; second, traditional treatment protocols focus on correcting coagulopathy with vitamin K1, thereby lacking a multidisciplinary collaborative intervention model for cases that involve urinary obstruction. Such deficiencies in understanding and treatment may lead to delays in diagnosis and treatment, and increase the risk of patients progressing to chronic kidney disease.

We present a case of 37 year old male with combined poisoning from brodifacoum (24.7 ng/mL) and bromadiolone (12 ng/mL). In this case, the patient developed blood clot obstruction in the bladder and urinary tract due to coagulopathy, which ultimately induced AKI under multifactorial effects. This case indicates that AKI caused by AR poisoning not only arises from classic glomerular hemorrhage resulting from coagulopathy, but also involves the superimposed effect of obstructive nephropathy. This finding enriches the understanding of renal injury related to AR poisoning and provides valuable information for clinical diagnosis and treatment. The aims of this study are to systematically describe the clinical characteristics, diagnostic workflow, and multidisciplinary treatment strategy for this rare mixed AR poisoning-induced AKI; to explore the potential pathogenic mechanisms underlying AKI caused by ARs; and to provide evidence-based references for clinicians to promptly recognize, diagnose, and manage similar complex cases, thereby reducing the risk of delayed treatment and progression to chronic kidney disease.

## Case presentation

On November 15, 2024, a 37-year-old previously healthy male patient presented to our hospital with chief complaints of “hematuria and gingival bleeding for 8 days, and anuria for 1 day.” On pre-admission day 8 (November 7), the patient developed painless macroscopic hematuria without obvious precipitating factors. The urine color progressively darkened from light red to dark red and contained small blood clots, along with spontaneous gingival bleeding. However, no medical intervention was sought. On pre-admission day 1 (November 14), the patient experienced acute urinary retention and periumbilical pain, prompting a visit to a local hospital. Abdominal ultrasonography performed at the local hospital revealed bilateral hydronephrosis and ureteral dilation. Despite empirical antibiotic treatment with levofloxacin and tranexamic acid for hemostasis at the local hospital, his symptoms showed no improvement. Consequently, he was transferred to our hospital on admission day (November 15). The patient, a farmer engaged in crop cultivation and poultry farming, denied any recent history of direct contact with or accidental ingestion of rodenticides.

**Figure 1:**
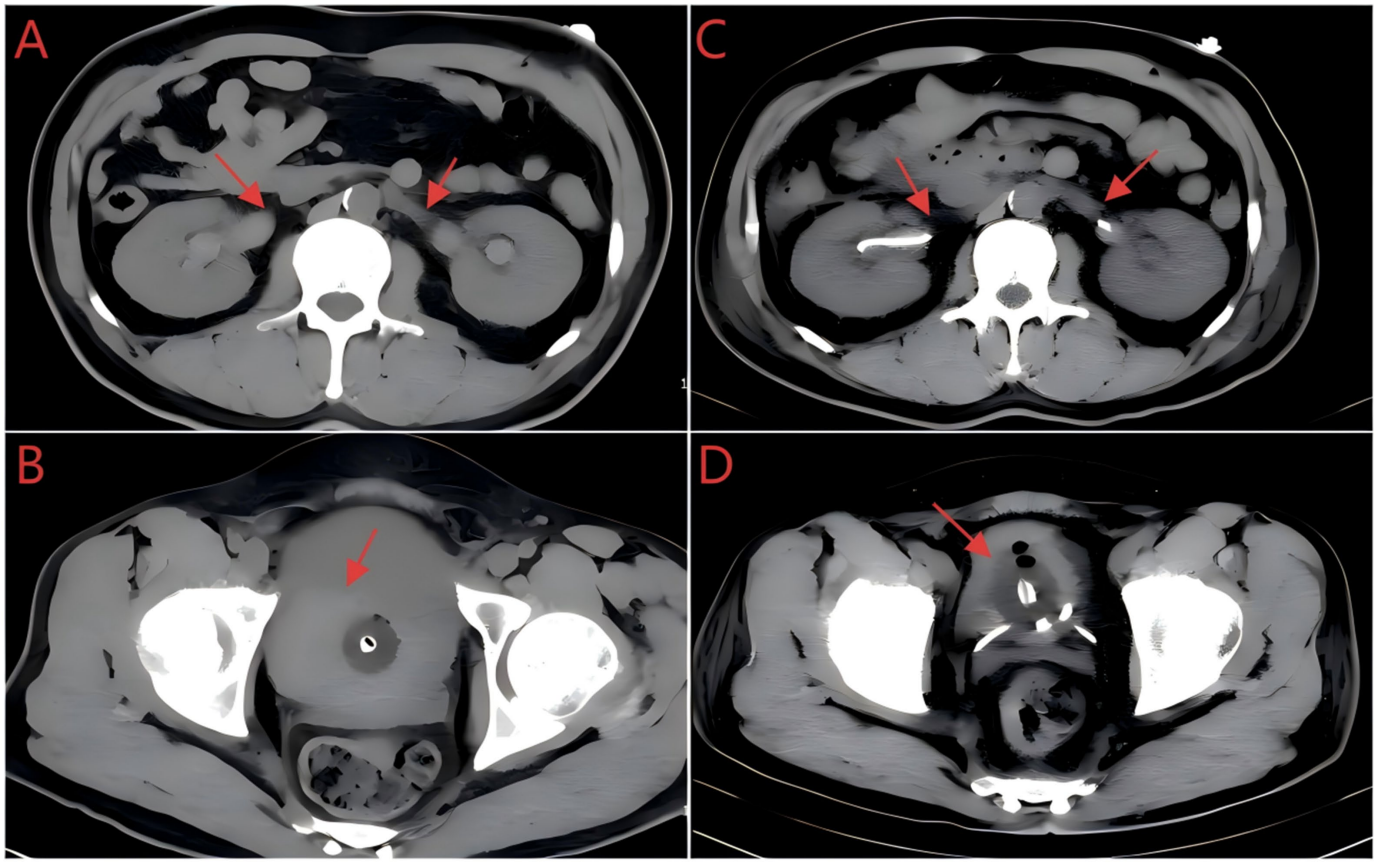
**(A)** Abdominal CT scan on admission (November 15, 2024) shows bilateral ureteral dilation with hydronephrosis, with red arrows indicating dilated ureters. **(B)** Abdominal CT scan on admission (November 15, 2024) reveals an overdistended bladder containing patchy hyperdense areas (red arrow) and an indwelling urinary catheter. **(C)** Repeat abdominal CT scan (November 17, 2024) demonstrates bilateral ureteral double-J stents *in situ* (red arrows), with mild dilation of bilateral renal pelvises and ureters. **(D)** Repeat abdominal CT scan (November 17, 2024) shows the indwelling urinary catheter within the bladder lumen, with residual small gas foci and minor patchy hyperdense areas.

Physical examination upon admission revealed a body temperature of 36.7 °C, pulse rate of 122 beats per minute, respiratory rate of 20 breaths per minute, and blood pressure of 115/76 mmHg. The patient exhibited pallor. Minor active bleeding was observed from the oral mucosa and gingiva. No costovertebral angle tenderness was elicited bilaterally. An indwelling urinary catheter was in place, draining dark red urine containing small blood clots. No other positive physical signs were identified. Relevant laboratory and imaging findings included: Complete blood count (CBC): white blood cell count (WBC) 14.53 × 10^9^/L, hemoglobin (Hb) 69 g/L, platelet count (PLT) 254 × 10^9^/L, and neutrophil percentage 84.90%; Procalcitonin (PCT) 0.29 ng/mL; Coagulation profile: prothrombin time (PT) > 120.00 s, international normalized ratio (INR) 13.58, activated partial thromboplastin time (APTT) 119.20 s, thrombin time (TT) 15.1 s, and fibrinogen (Fib) 6.12 g/L; Coagulation factor: Factor II 23.4%, Factor VII 43.5%, Factor IX 42.9%, Factor X 22.2%; Renal function: serum creatinine (Scr) 1.86 mg/dL; Abdominal computed tomography (CT) scan showed bilateral ureteral dilation with hydronephrosis, an overdistended bladder containing patchy hyperdense areas and a small amount of intraluminal gas, with an indwelling urinary catheter in place (Figures 1A,B). Following admission, immediate interventions included intravenous infusion of 400 mL leukocyte-depleted suspended red blood cells to ameliorate anemia, alongside intravenous infusion of 200 mL fresh frozen plasma combined with vitamin K1 (initial dose 80 mg, maintenance dose 20 mg every 12 h) to improve coagulation function.

On post-admission day 1 (November 16), repeat investigations showed: CBC: WBC 13.97 × 10^9^/L, Hb 60 g/L, PLT 211 × 10^9^/L, neutrophil percentage 75.70%; Coagulation profile: PT 12.70 s, INR 1.16, APTT 30.50 s, TT 16.90 s, Fib 4.90 g/L; Renal function: Scr 5.93 mg/dL; Blood toxicology screen detected brodifacoum 24.7 ng/mL and bromadiolone 12 ng/mL. Comprehensive assessment diagnosed rodenticide poisoning. Although coagulation function showed some improvement with treatment, the urine output on the day of admission was only 35 mL. Combined with the abdominal CT findings, the diagnosis of obstructive nephropathy secondary to ureteral and bladder blood clots was established. After ineffective continuous bladder irrigation, cystoscopic evacuation of blood clots and bilateral ureteroscopic double-J stent placement were performed on post-admission day 1. An additional 400 mL of leukocyte-depleted suspended red blood cells was transfused intravenously to improve the anemic state. The total urine output on November 16 recovered to 300 mL. Repeat testing on post-admission day 2 (November 17) showed Scr 5.64 mg/dL. Repeat abdominal CT showed a postoperative status following bilateral ureteral double-J stents placement, with the stents *in situ*, demonstrating mild dilation of bilateral renal pelvises and ureters, slightly increased intraluminal density, an unfilled bladder, an indwelling urinary catheter within the bladder lumen, and a small amount of gas with small patchy hyperdense areas within the bladder (Figures 1C,D). Given the persistent renal injury and volume overload, continuous renal replacement therapy (CRRT) was initiated from 13:00 on post-admission day 2 and continued until 10:40 on post-admission day 3 (November 18). On post-admission day 4 (November 19), the patient’s urine output increased to 2,580 mL. Investigations on post-admission day 5 (November 20) showed: CBC: WBC 8.39 × 10^9^/L, Hb 92 g/L, PLT 331 × 10^9^/L; Coagulation factors: Factor II 67.7%, Factor VII 112.1%, Factor IX 106.6%, Factor X 66.3%; Urinalysis: protein 2+, occult blood 3+, microscopic red blood cells 3+/HPF, red blood cell morphology: normocytic red blood cells, red blood cell fragments and crenated red blood cells; Coagulation profile: PT 12.6 s, INR 1.15, APTT 26.3 s, TT 16.2 s, Fib 5.35 g/L; Renal function: Scr 1.52 mg/dL.

**Figure 2:**
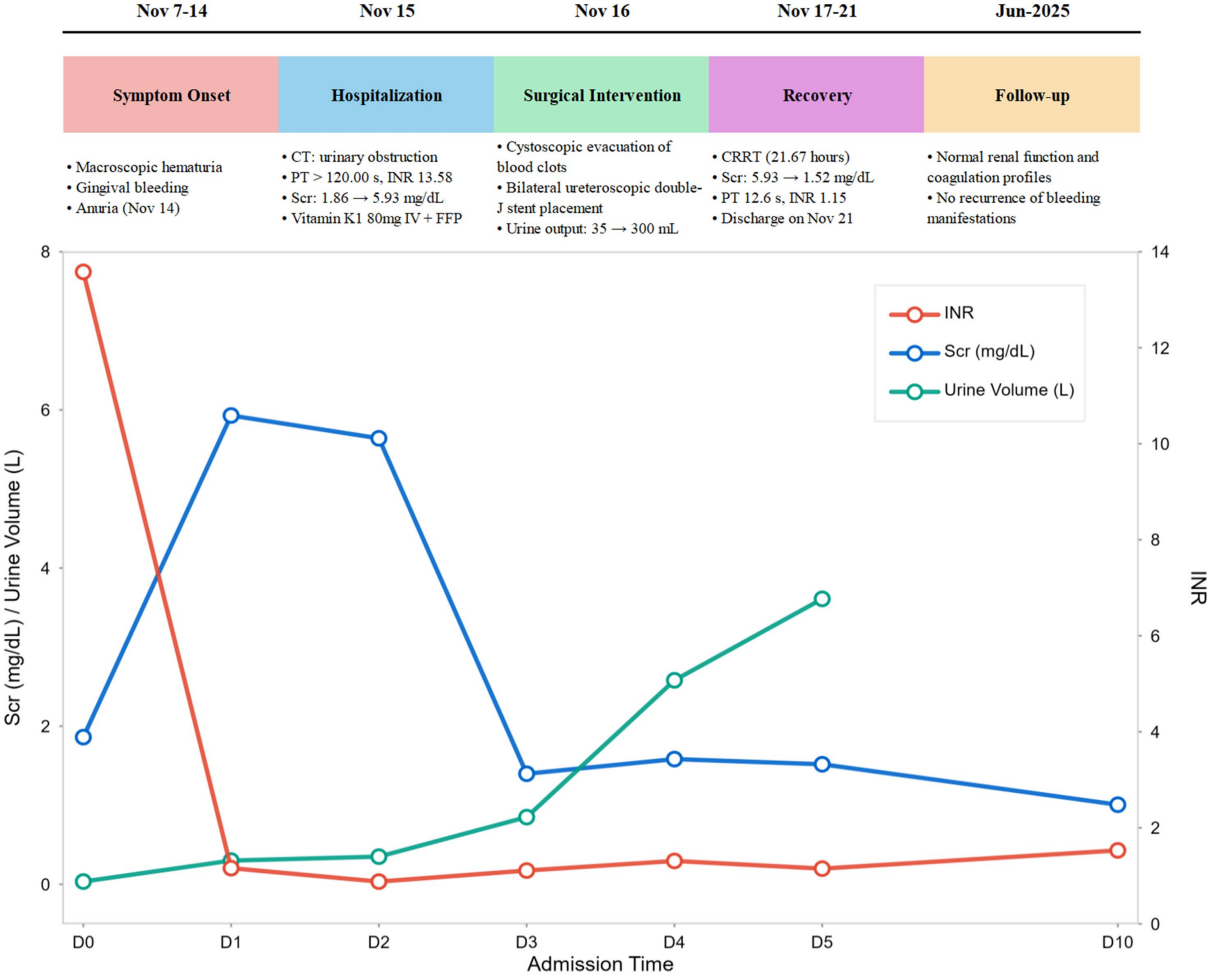
Case progress timeline.

The patient was discharged on post-admission day 6 (November 21). After discharge, the local hospital prescribed oral vitamin K1 (40 mg once daily) to correct coagulation function. Follow-up on post-admission day 10 (November 25) showed coagulation profile: PT 11.1 s, INR 0.88, APTT 28.7 s, TT 13.4 s, Fib 5.05 g/L; Renal function: Scr 1.01 mg/dL. Telephone follow-up at 7 months post-discharge (June 20, 2025), revealed that the patient had continued oral vitamin K1 until February 20, 2025. During this period, repeat renal function tests and coagulation profiles were normal, with no recurrence of hematuria or bleeding manifestations in other organs. The timeline illustrating progress of the case is shown in Figure 2. Key laboratory values during hospitalization and follow-up are summarized in Table 1.

**Table 1:** Key laboratory examinations.

Parameters	Reference range, this hospital	Day 0 (Admission)	Day 1	Day 2	Day 3	Day 4	Day 5	Day 10 (follow-up)
Coagulation profile								
Prothrombin time (s)	10–14	>120	12.7	10.3	12.2	14.2	12.6	11.1
International normalized ratio	0.8–1.21	13.58	1.16	0.88	1.11	1.31	1.15	0.88
Activated partial thromboplastin time (s)	24.8–33.8	119.2	30.5	31.57	27.3	29.6	26.3	28.7
Thrombin time (s)	14–21	15.1	16.9	18.7	16.1	17.6	16.2	13.4
Fibrinogen (g/L)	1.8–3.7	6.12	4.9	6.25	5.54	5.93	5.35	5.05
Coagulation factor								
II (%)	70–120	23.4	–	–	–	44.1	67.7	–
VII (%)	70–120	43.5	–	–	–	39.1	112.1	–
IX (%)	70–120	42.9	–	–	–	64.6	106.6	–
X (%)	70–120	22.2				47.3	66.3	
Complete blood count								
Hemoglobin (g/L)	130–175	69	60	86	94	–	92	121
Platelet count (×10^9^/L)	125–350	254	211	247	320	–	331	290
Renal function								
Urea (mmol/L)	3.2–7.1	12.82	19.08	20.1	5.34	–	11.24	7.5
Serum creatinine (mg/dL)	0.66–1.24	1.86	5.93	5.64	1.40	–	1.52	1.01
Urine								
Urine color	Clear to yellow	Red	Red	Red	Tea-colored	Tea-colored	Clear to yellow	Clear to yellow
Urine volume (mL)	1,000–2,500	35	300	350	850	2,580	3,610	–

## Discussion

Brodifacoum and bromadiolone are long-acting ARs that share a similar mechanism of action with warfarin. All these agents disrupt the vitamin K cycle through inhibition of vitamin K epoxide reductase (VKOR), which in turn impairs the *γ*-carboxylation of coagulation factors II, VII, IX, and X, leading to the development of acquired vitamin K-dependent coagulopathy (AVKDC) (5, 9–11). In this case, the patient manifested not only the typical symptoms of AVKDC, including hematuria and gingival bleeding, but also AKI. While previous reports have frequently linked AR-associated AKI to anticoagulation-related nephropathy (ARN) (8), abdominal CT in this case showed patchy hyperdense areas in the overdistended bladder, together with bilateral ureteral dilation and hydronephrosis. This confirms that, apart from ARN, mechanical obstruction caused by blood clots in the bladder and ureters also contributed to the development of AKI. This finding revises the traditional view that renal injury induced by ARs arises solely from glomerular bleeding, and offers a new angle for the clinical evaluation of renal injury mechanisms in patients with AR poisoning. To our knowledge, this represents the first reported case of AKI mediated by a dual mechanism secondary to AR poisoning.

Regarding the mechanism of AKI occurrence, this case demonstrates the synergistic effect of direct toxicity and indirect damage. Regarding direct renal toxicity, although research on the direct effects of bromadiolone and brodifacoum on renal tubular epithelial cells remains limited, lipophilic toxins may accumulate in renal tubular epithelial cells via the bloodstream, thereby disrupting mitochondrial function or inducing oxidative stress—particularly when toxin concentrations surpass hepatic and renal metabolic thresholds, which may exacerbate tubular cell injury. The indirect damage mechanism is intricately linked to the classic anticoagulant effect of coumarin-type toxins. Severe coagulopathy, analogous to the pathogenesis of ARN, can lead to bleeding in Bowman’s capsule and renal tubules. The presence of red blood cells in the tubules causes luminal obstruction, resulting in local ischemia and tubular occlusion (12). In addition to obstruction, breakdown products of red blood cells (e.g., free hemoglobin and hemosiderin) directly damage the tubules via oxidative stress (13). Furthermore, blood clots from local urinary tract hemorrhage induce ureteral and bladder obstruction, while coagulation-induced renal microthrombosis exacerbates renal ischemia and hypoxia. These combined mechanisms ultimately form a cascade of “coagulation disorder → glomerular hemorrhage → urinary obstruction → renal microthrombosis,” leading to rapid renal function deterioration. It should be noted that, given the significant coagulopathy in the patient of this case, the risk of bleeding associated with renal biopsy was relatively high; therefore, renal biopsy was not performed. As a result, histological confirmation is lacking to clarify the extent of renal tubular injury and the details of glomerular hemorrhage. However, based on comprehensive multi-dimensional evidence, the diagnosis of AKI caused by the dual mechanisms of ARN and obstructive nephropathy can still be confirmed: Laboratory investigations revealed significant coagulation abnormalities, reduced activity of vitamin K-dependent coagulation factors, and progressive elevation of Scr, while blood toxicology analysis detected brodifacoum and bromadiolone, confirming the etiological factor; Abdominal CT showed bilateral ureteral dilation with hydronephrosis and patchy hyperdense areas in the bladder, which were consistent with the manifestations of urinary tract obstruction caused by blood clots; After treatment including correction of coagulopathy, cystoscopic evacuation of blood clots, and bilateral ureteroscopic double-J stent placement, the patient’s Scr decreased and urine output returned to normal, and the therapeutic response further verified the accuracy of the diagnosis.

Timely intervention and multidisciplinary collaboration are vital in managing this case. Standard coagulopathy correction for AR poisoning involves combined vitamin K1 and fresh frozen plasma administration (5). Vitamin K1 competitively inhibits AR binding to VKOR, thereby promoting coagulation factor synthesis; fresh frozen plasma rapidly replenishes deficient factors and corrects bleeding tendency. For severe or refractory poisoning cases that show no improvement with standard treatment, hemoperfusion can be used as an adjuvant therapeutic option to assist in the removal of excessive toxins from the body, thereby improving treatment response (14). Here, vitamin K1 and fresh frozen plasma were administered immediately on admission, with marked coagulation improvement observed the next day, confirming treatment efficacy. For urinary obstruction, cystoscopic clot removal and double-J stent placement are key interventions. Large bladder clots cause urethral obstruction, limiting conservative treatment efficacy. Direct cystoscopic clot evacuation with ureteral stenting effectively restored urinary flow. Postoperatively, urine output increased substantially and renal function improved, validating this strategy. Additionally, timely CRRT initiation for renal injury and volume overload maintains internal milieu stability and promotes renal recovery. Appropriate timing and indications critically influence prognosis.

A review of the literature (8, 15, 16) suggests that rodenticide poisoning cases typically affect a single system. For instance, patients may present as organ bleeding due to coagulopathy alone, or mild renal injury attributable to the ARN mechanism. In contrast, this case demonstrates that AR poisoning can lead to both severe coagulopathy and renal injury, where the latter arises from dual mechanisms of ARN and obstructive nephropathy. This supports that clinicians managing AR-poisoned patients should routinely perform urinary system ultrasound or CT scans. Combined with coagulation and renal function monitoring, this allows prompt identification of obstruction and accurate diagnosis of renal injury causes.

In maintaining vitamin K1 therapy, considering the long half-life pharmacokinetic characteristics of brodifacoum and bromadiolone (17), vitamin K1 maintenance therapy requires individualized regimens. Current evidence lacks consensus on dosage and duration. A review of the literature indicates that the maintenance dose of vitamin K1 ranges from 15 to 600 mg per day, with the most commonly prescribed dosage being 100 mg per day, and the average treatment duration is 168 days. Additionally, concerning the duration of vitamin K1 therapy, most ARs are metabolized in the liver and excreted in urine, with a small fraction excreted unchanged; however, the excretion rate is slow, with a release time spanning from 3 months to 1 year, posing a risk of recurrent poisoning (18). Therefore, it is recommended that the concentration of ARs be quantified weekly, and vitamin K1 should be continuously administered until the ARs concentration falls below 10 ng/mL (19, 20). Furthermore, clinicians should adjust the treatment dosage of vitamin K1 based on coagulation function indicators (such as PT and INR) to mitigate the risk of rebound coagulopathy due to residual toxins. With respect to renal function monitoring, given the potential risk of progression from AKI to chronic kidney disease, regular monitoring of Scr, urinalysis, and urinary microalbumin every 1–2 months is advised to identify early signs of subclinical injuries, such as tubulointerstitial fibrosis. From a preventive perspective, this case underscores the critical need for strengthening the standardized management of rodenticides within the community. Strict control over rodenticide sales channels, regulation of usage instructions, and increasing public awareness of their toxicity are essential to prevent poisoning incidents due to accidental ingestion or contact.

## Conclusion

In conclusion, this case supplies substantial evidence for the multifactorial pathogenesis of AKI induced by AR poisoning. Through systematic analysis and multidisciplinary treatment implementation, it reinforces the need for prompt identification of urinary obstruction in AR-induced AKI patients and highlights interdisciplinary collaboration value in complex case management, thereby offering practical clinical guidance. Nevertheless, this report has limitations. Absence of renal biopsy obscures direct toxin-induced renal pathology. Additionally, the lack of post-discharge blood concentration monitoring for brodifacoum and bromadiolone represents a significant limitation in comprehensive assessment and may affect the optimization of treatment duration. Future studies should prioritize investigating AR renal toxicity mechanisms and developing toxin-mediated AKI risk stratification models. Such work will establish a stronger theoretical basis for targeted interventions, to advance diagnostic and therapeutic approaches in this field.

## Data Availability

The raw data supporting the conclusions of this article will be made available by the authors, without undue reservation.
